# IDOMAL: an ontology for malaria

**DOI:** 10.1186/1475-2875-9-230

**Published:** 2010-08-10

**Authors:** Pantelis Topalis, Elvira Mitraka, Ioana Bujila, Elena Deligianni, Emmanuel Dialynas, Inga Siden-Kiamos, Marita Troye-Blomberg, Christos Louis

**Affiliations:** 1 Institute of Molecular Biology and Biotechnology, Foundation for Research and Technology-Hellas, 700 13 Heraklion, Crete, Greece; 2 Department of Biology, University of Crete, 711 10 Heraklion, Crete, Greece; 3 Department of Immunology, Stockholm University, SE-106 91 Stockholm, Sweden

## Abstract

**Background:**

Ontologies are rapidly becoming a necessity for the design of efficient information technology tools, especially databases, because they permit the organization of stored data using logical rules and defined terms that are understood by both humans and machines. This has as consequence both an enhanced usage and interoperability of databases and related resources. It is hoped that IDOMAL, the ontology of malaria will prove a valuable instrument when implemented in both malaria research and control measures.

**Methods:**

The OBOEdit2 software was used for the construction of the ontology. IDOMAL is based on the Basic Formal Ontology (BFO) and follows the rules set by the OBO Foundry consortium.

**Results:**

The first version of the malaria ontology covers both clinical and epidemiological aspects of the disease, as well as disease and vector biology. IDOMAL is meant to later become the nucleation site for a much larger ontology of vector borne diseases, which will itself be an extension of a large ontology of infectious diseases (IDO). The latter is currently being developed in the frame of a large international collaborative effort.

**Conclusions:**

IDOMAL, already freely available in its first version, will form part of a suite of ontologies that will be used to drive IT tools and databases specifically constructed to help control malaria and, later, other vector-borne diseases. This suite already consists of the ontology described here as well as the one on insecticide resistance that has been available for some time. Additional components are being developed and introduced into IDOMAL.

## Background

The failure of the campaign to eradicate malaria about 40 years ago led, among others, to a widespread notion that this disease can simply not be wiped out. This modified the goals of the majority of malaria workers worldwide towards achieving a mitigation of the problem, rather than seeking a final solution. On the other hand it is evident that campaigns based both on novel and traditional concepts, have been highly successful; the key example is the European paradigm of malaria eradication. Moreover, the advent of modern molecular biological techniques, today ranging into genomics and post-genomics, have also provided an impetus towards the development of original and groundbreaking approaches. For example, on the level of malaria entomology, an increased understanding of vector biology in areas such as genetics, molecular and population biology has formed the basis for the design of potential future anti-malarial strategies: these are to be based on the use of genetically modified mosquitoes in order to accomplish a (permanent?) break of transmission cycles.

The recent resurrection of the idea of malaria eradication attributed to Melinda and Bill Gates [1] and immediately adopted by many malariologists, even if only as a "distant dream" [see [2,3]], has moved many research efforts towards schemes aiming at this ultimate goal. The relative optimism with which such a possibility was met was based, among others, on a series of realities that differentiate the present situation from that of the second half of the previous century. These facts primarily include the increased knowledge on all aspects of the biology of the disease, and most importantly, the availability of tools that, fifty years ago, could only be found in the realm of science fiction. Modern information technology (IT) and logistics are good examples of this.

Bioinformatics, as a specialized and logical descendant of computer sciences and IT, evolved mainly due to the development of DNA sequencing and the need to access and understand those primary data. It received its first boost through automated sequencing and it has progressed even more in order to be able to handle the immense accrual of information that keeps accumulating through genomics in the widest sense. In parallel to the actual sequence analysis, a major part of bioinformatics deals with the development and maintenance of databases in terms of, among others, the organization of their contents, their accessibility, and the cross-talk between them.

It was recently suggested to use ontologies as an efficient instrument to enhance the impact of IT tools in vector biology and malaria entomology [4,5]. This can be achieved by building databases and/or decision support systems driven by wide-ranging ontologies that follow common and established rules. In information science an ontology is a formal representation of the knowledge, which includes the definition of concepts within a given domain as well as the relations between these concepts. In a simplified example, a given biomedical ontology would provide the definition of the term "translation", list its synonym "protein synthesis", and also include its parent (e.g. biological process, metabolic process, gene expression, etc.) and child terms (e.g. initiation, elongation, termination, tRNA aminoacylation, etc.). All of these terms and the relations (in our examples, "*is_a" *and "*part_of*" relations) are well understood by humans but also, most importantly, all computers that have adopted the usage of a given ontology. Although an ontology is often confused with a controlled vocabulary, the latter does not usually use relations and, thus, looses power in terms of computer use: For example, a search of a database driven by the example ontology just mentioned would list, in searches using the string "translation" all items annotated with the term "elongation", since it would be known that the former is a parent term of the latter. It is apparent that if this kind of data exchange and comprehension by information systems can be achieved, a world-wide malaria eradication campaign would greatly benefit from the adoption of standardized ontologies, which would allow for an extensive data exchange across national boundaries and specific projects.

The power of such biomedical ontologies can be best exemplified through the immense success of the Gene Ontology (GO) [6], that has not only allowed improved annotation of experimental data but which, concomitantly, led to an easier comprehensive data mining as well as understanding of molecular biology. VectorBase [7], the database of genomic information on disease vectors, therefore recently incorporated a section on insecticide resistance (IRbase) that fully relies on a specially designed ontology called MIRO [8]. Here, the first version of an ontology for malaria called IDOMAL (Infectious Disease Ontology-MALaria) is described; it is made publicly available in order to seek feedback from the wide community of malariologists.

## Methods

The OBOEdit2 software [9], which is freely available for downloading [10] was used for the construction of the IDOMAL. The malaria ontology is based on BFO, the Basic Formal Ontology [11,12] and it follows, in full, the rules set by the OBO Foundry consortium [13]. IDOMAL can be downloaded from Vectorbase [14], and it can be viewed and browsed on line at the NCBO bioportal [15].

## Results and Discussion

### IDOMAL: the format and the contents

A decision to build an ontology immediately raises some crucial questions that should be answered at the very beginning of the project. Perhaps the first one is the question concerning the primary reasoning that led to the initiation of a project: what is the real need for a given ontology? In the case of the IDOMAL it was clear that there is a vast wealth of knowledge available that could be put to use for the purpose of malaria control by malaria experts, database developers and technicians constructing decision support tools for the disease. Unfortunately, though, the data that range back several decades have been annotated using a multitude of different criteria. This makes it tedious to "unify" the information in order to exploit it to the maximum. We therefore decided to develop a global malaria ontology having in mind two pre-requisites a) the ontology will aim at maximum interoperability and b) it will be amenable to future expansion to encompass aspects that would not be part of it in the initial versions. For several reasons that will be laid out below, it was decided to construct the ontology in the frame of IDO, the Infectious Disease Ontology [16], a loose consortium of research groups aiming at developing ontologies for a variety of infectious diseases that include brucellosis, Dengue fever, infective endocarditis, influenza, tuberculosis and others. Within this consortium, it was decided to initiate the implementation of the project of vector-borne disease ontologies with malaria, indisputably the most important vector-borne disease for global health.

**Figure 1:**
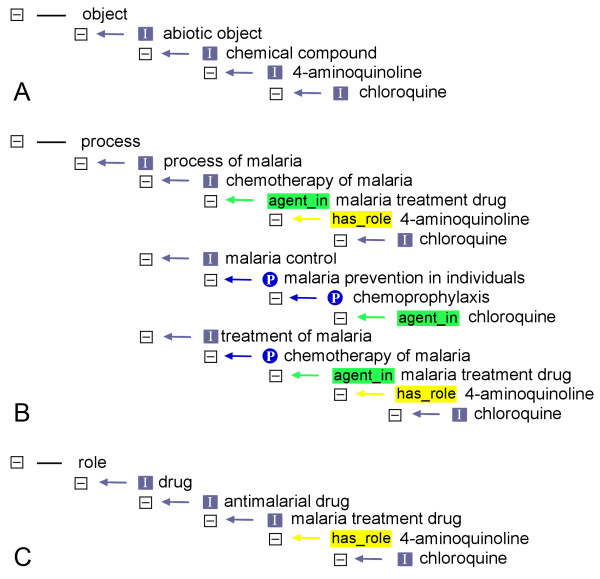
**The position of *chloroquine *in IDOMAL**. The figure shows the positions of *chloroquine *in the IDOMAL ontology tree. A: the *is_a *path from *chloroquine *to the top-level *object *class; B: three different paths leading from *chloroquine *and converging to *process of malaria *(an *is_a *child of the top-level class *process*) linking parent-children terms with different relations; C: the third path of *chloroquine *leading to the top-level class *role *through two different relations. The different relations are indicated by different signs and colours: grey box = *is_a*, blue circle = *part_of*, yellow box = *has_role*, green box = *agent_in*.

**Figure 2:**
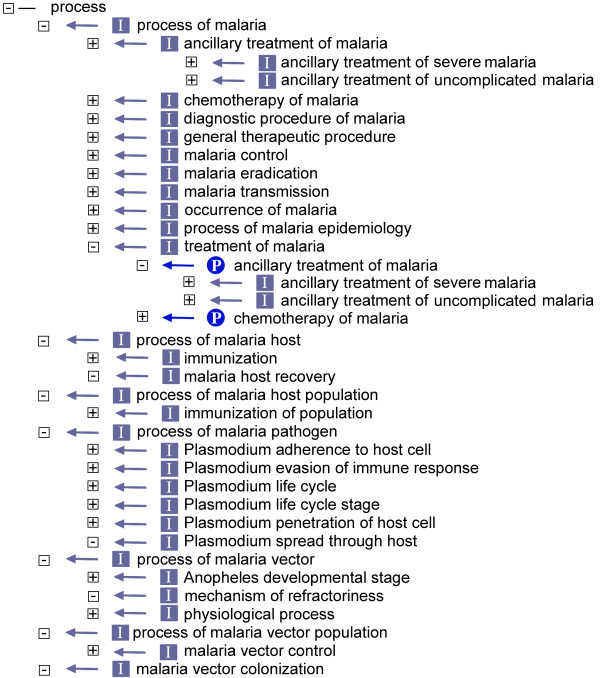
**The class *process *in IDOMAL**. The upper-most terms within the class *process *are indicated here. Most relations shown are *is_a *relations (grey box) with two exceptions in which *part_of *relations (blue circles) are indicated. The small boxes containing a plus sign signify that the term contains a number of children terms, while the boxes with a minus sign show a "terminal" term that has no children in the ontology.

IDO will be a top-level ontology that will form the neutral core for all other sub-domain-specific ontologies to be developed as disease-specific extensions of the core. In this sense, IDO will function similarly to the CARO, the Common Anatomy Reference Ontology [17], that is the nucleus for many anatomical ontologies, and which also served as the basis for the two ontologies built by our group for the anatomy of arthropod disease vectors, TGMA for mosquitoes and TADS for ticks [18]. Similar to the CARO, it has been decided to base IDO and all its "components" on BFO [11,12]. Being domain neutral, BFO allows for a unified treatment of all biomedical items that are to be described in different ontologies. The BFO structure was therefore exploited to incorporate in IDOMAL parts of the previously developed insecticide resistance ontology MIRO [8] without having to go through its intricate restructuring (see below). Given the dependence of IDOMAL on BFO, its architecture and the details of its structure may not be immediately decipherable by non-experts. For example, not only are the distinctions between *process *and *fiat process part *(a processual entity that is part of a process but that does not have *bona fide *beginning and ending that correspond to real discontinuities [19]) or between *disposition*, *state *and *condition *hard to recognize for the uninitiated, but the overall "architecture" may seem complicated in spite of being ontologically correct. The example chloroquine, outlined in figure 1, illustrates this point. The term is found five times in the ontology, once as an *object*, three times as a child of the class *process *and once as a child of the class *role*. Of these five times, only one links chloroquine to the top level, *object*, using a complete *is_a *relation path (figure 1A). There are many similar cases in IDOMAL; a second example can be found within the class *process/process of malaria *where, at a high level, the term *ancillary treatment of malaria *can be identified, containing the terms relating to both severe and uncomplicated malaria. A sibling to this term, though, is *treatment of malaria*, which itself contains the mentioned term *ancillary treatment of malaria*, again with all of its children. Although this may seem illogical at first glance, this is not so: in the former case the indispensable *is_a *path is set up while in the latter the parthood relation is described. Figure 2 shows the contents of the *process *class illustrating the example mentioned while Table 1 shows a summary of the overall contents of the ontology, and the top-level classes that they have been ontologically attributed to. This table is obviously only a schematic, summary representation.

**Table 1:** The upper classes of IDOMAL

Class	Number of terms	Contents summary
condition	45	clinical features of malaria host (e.g. symptoms and signs, etc.)
disposition	77	infectious disease (malaria - > transmission, progression - > clinical manifestation, etc.
fiat process part	121	mostly vector-related "processes"
object	1148	a) abiotic objects (chemical compounds, including insecticides, antimalarials), screening material, environmental/geographic features, etc.
		b) biotic objects (anatomical structures, host -, vector - and parasite species, etc.)
object aggregate	89	populations (host, vector, parasite), protein complexes
process	1320	processes of malaria, host, vector, parasites, populations, combination therapy, diagnostic tests, etc.
process boundary	2	
quality	253	qualities of malaria, environment, host, vector, parasites and populations
role	576	roles of biological and chemical substances (e.g. drugs, enzymes, factors, etc.), parasites, breeding sites
spatiotemporal region	6	
temporal region	5	

The table lists the upper classes of the ontology and includes, only for the heavily populated ones, a summary of the main contents of the class. The numbers denote the number of terms found in each one of the classes.

It should be stressed here that as the ontology is a specialized tool and not a simple "dictionary", the immediate advantages of the BFO representation, central for database development, not being immediately noticeable when browsing through the IDOMAL. At this time (version 1.2) it contains 2392 unique terms of which 2377 (> 99.3%) are defined. These terms are distributed in 12 upper level classes, all defined by BFO. Table 1 also shows the number of individual descendants for each of these classes in IDOMAL. The two classes dealing with processes, *process *and *fiat process part*, are made out of a total of 1441 descendants, whereby the latter class contains, almost exclusively, vector-related terms that are listed in Table 2. Another densely populated class is "object" (1148 terms); this is due, on one hand, to the inclusion of such terms as a comprehensive list of hosts, parasites and malaria vectors, all of them under "biotic", and on the other, to the inclusion of "chemical compounds" that includes extensive lists of anti-malarial drugs, insecticides and several proteins, again, from host, parasite and vector. A "similar" class is *object aggregate*, which, among others such as populations-related terms, also lists drug combinations and diagnostic tests. *quality *and *role *are two additional heavily populated classes (253 and 576 terms respectively), while the other classes are presently not very densely populated. Obviously, the total numbers of descendants of the top classes (3699) don't add up to the total number of terms listed in the ontology; the reason for this is that in addition to being connected to their parent directly through an *is_a *relation, several terms are also equally connected to other terms through relations of a different kind such as, for example, *part_of*, *realizes*, *preceded_by *and others, similar to the example illustrated previously in figure 1. Finally, no indications as to the contents for some of the upper classes were included in Table 1; the reason for this is the scarce population with terms. For example, "state" contains only two terms, oostasis and diapause, while "spatiotemporal region" lists the five developmental gates described for follicular development in mosquitoes.

**Table 2:** Physiological processes and "fiat process parts" of malaria vector listed in IDOMAL

*process*		*fiat process part*	
behavioural process	189	cell-to-cell communication	0
chorion formation	2	descent to the body surface and alighting	4
circulation	0	descent to water surface	0
developmental process	30	development of competence	0
distension of midgut	6	digestion of food	27
egg laying	1	equilibrium during flight	0
endocrine system process	1	exploration and examination of body surface	33
excretion	15	flight orientation	0
fertilization	0	food ingestion	6
formation of ovarian follicles	0	formation of assembly	9
formation of peritrophic matrix	2	gliding	0
growth	4	hovering	0
immune system process	17	internalization of vitellogenin	1
muscular system process	25	long-range approach	30
nervous system process	5	organelle synthesis in midgut cells	5
nutritional process	1	ovarian cycle	27
previtellogenic development	2	ovarian developmental stages (Christophers)	10
regulation of biological process	1	ovarian developmental stages (Troy et al.)	9
release of 20-hydroxyecdysone	0	oviposition	0
reproduction	90	persistent locomotion	0
respiration	5	process of oogenesis	40
response to stimulus	87	process of ovulation	1
rRNA synthesis in oocyte and nurse cells	0	production of digestive enzymes	4
saliva secretion	0	senses and flight response during mating	29
secretion of peritrophic matrix in larvae	0	short-range approach to the host	5
sensory perception	21	skin-hopping	0
stimulation of vitellogenin synthesis	0		
termination stage	0		
untrastructural change in the trophocyte	6		
vector metabolic process	32		
vitellogenesis	10		
vitellogenic stage	1		
vitellogenin synthesis	2		

The table lists, alphabetically, physiological processes and fiat process parts of malaria vectors that are currently listed in IDOMAL. The numbers refer to the numbers of individual child terms of a given term. When a zero (0) is indicated, the term in the table has no children listed in the ontology.

### IDOMAL: the disease-related terms

Terms pertinent to the malaria disease as such relate to several distinct aspects of malaria. These obviously include clinical manifestations, therapeutic approaches and epidemiology, but also terms that relate to *Plasmodium *parasites as aetiologic agents. As the aim of IDOMAL is not to build a general disease ontology, the contents focus on terms that are pertinent to malaria as such; nevertheless they are quite complete, aiming at annotating, when need is, all aspects of clinical malaria. Among others, the ontology lists the generic names of all currently available anti-malarial drugs (proprietary names are often listed as synonyms) and commonly used combination therapies; all available diagnostic procedures, including all available rapid diagnostic tests (RDT, as of 2008); therapeutic approaches, including ancillary treatment of malaria. It should be stressed at this point that terms that are already described, defined and given a separate ID number by a higher order ontology such as, for example, IDO or another generic and publically available (open) biomedical ontology, may in the future replace, in full or in part, some of the terms used in IDOMAL. Should this be the case, obviously, the current ID numbers will be kept as cross-references and terms with a slightly different wording may also be kept as synonyms. A good example for this are the *Anopheles *breeding sites (ontologically: roles!), which have been described by, and are already listed in IDOMAL with the ID of ENVO, the Environment Ontology [20].

In addition to clinical aspects of malaria one additional feature that is also dealt with in IDOMAL is disease biology, including immunology. Here, we were faced with the choice of describing several terms in the ontology in detail or of handling them on a shallow level and relying on a future database for their potential detailed "description". The best cases in point for this are the proteins that have been described as being involved in different crucial host-vector interactions. One example can demonstrate the question faced, as well as the possibility to tackle its solution.

**Figure 3:**
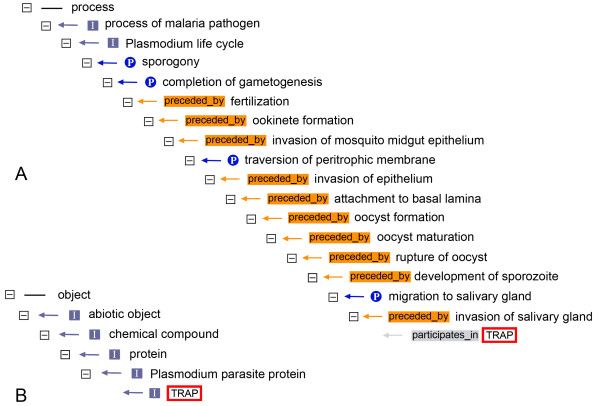
**The TRAP protein in IDOMAL**. The two different paths in which the protein TRAP (enclosed in a red and white rectangle) is found are shown here. In B, the *is_a *path leads to the upper class *object*, while in A the position of TRAP in the clade leading to the class *process *is shown. In addition to *is_a *(grey rectangle) and *part_of *(blue circle), two additional relations are used for the correct setting of the term: *participates_in *(grey rectangle) and *preceded_by *(orange rectangle).

The thrombospondin-related anonymous protein (TRAP) from *Plasmodium *was first identified more than 20 years ago in the human parasite *P. falciparum *[21], and since then in several more *Plasmodium *species. The function of TRAP, a sporozoite transmembrane protein, is to interact with the substrate in the process of motility [22,23]. Moreover, it was later found that TRAP plays an active role in invasion of hepatocytes [24,25]. Should TRAP be included in the malaria ontology as a term? To begin with, there are several *Plasmodium *species for which there is complete lack of information on the respective protein and the gene that encodes it; these species include, unfortunately, even human parasites. Furthermore, specific information on already identified genes/proteins is often stored in databases such as PlasmoDB [26], and so far no need for an annotation in terms of the TRAP name has surfaced or, at least, no such need is described in any major publication. These facts, therefore, would imply that a protein such as TRAP should not be included in a malaria ontology. On the other hand, TRAP has been discussed as a potential vaccine [27,28]. Thus, in a database that deals with vaccines, it is possible that a generic TRAP term might be needed for potential annotation. Similar thoughts concerning other *Plasmodium *proteins which could potentially become pharmacological targets have led us to the inclusion in IDOMAL of TRAP and several more proteins that are potentially involved in vector-parasite and host-parasite interactions. Figure 3 shows the term *TRAP *in two clades of the ontology, a longer one describing the protein in its biological context using four different relations (A, top right) and a short one showing the *is_a *path from *TRAP *to its uppermost parent class in five steps (B, bottom left). At this moment IDOMAL lists 86 *Plasmodium *proteins but, obviously, the number of such malaria-related molecules will certainly increase in the future as knowledge on the molecular biology of malaria increases and several more will have to be added to IDOMAL.

Similar considerations are valid for terms dealing with malaria immunology, in general, and malaria vaccines, in particular. The rapid progress achieved in these fields, combined with the complicated immunological aspects of the disease [29,30] are principally to blame for an initial relative scarcity of relevant information in IDOMAL. It is noted, though, that attention was focused on immunology-related terms that are "linked" to processes of malaria and not immunity in general, and certainly no description of the immune response in vectors is described yet. Table 3 lists all vertebrate host proteins that are currently listed in the ontology. Several more terms relating to host immunity can be found in both *process of malaria host *and *quality of host*. As stated above, these terms don't include important, yet malaria-unrelated entities.

**Table 3:** Malaria-related vertebrate host proteins listed in IDOMAL

C3b			
CD36			
complement receptor 1			
defensin			
granzyme B			
human actin			
human ankyrin			
human band 3 protein			
human band 4.1 protein			
human Duffy blood group antigen			
human glucose-6-phosphate dehydrogenase			
human glycophorin A			
human glycophorin C			
human haemoglobin			
	variant haemoglobin		
		haemoglobin C	
		haemoglobin E	
		haemoglobin S	
		thalassaemia-related haemoglobin	
			alpha thalassaemia-related haemoglobin
			beta thalassaemia-related haemoglobin
	wild type haemoglobin		
human spectrin			
immunoglobulin			
	immunoglobulin E		
	immunoglobulin G		
		immunoglobulin G1	
		immunoglobulin G3	
	immunoglobulin M		
interferon gamma			
interleukin 10			
interleukin 12			
interleukin 13			
interleukin 2			
interleukin 4			
lysozyme			
perforin			
toll like receptor 2			
toll like receptor 9			
tumor necrosis factor-alpha			

The table lists, alphabetically, all malaria-related vertebrate host proteins that are currently listed in IDOMAL. Proteins that are found tab-shifted rightwards in any line of the table are *is_a *children of the respective higher order term.

Finally, the ontology evidently includes a series of terms that pertain to the parasite and its role as a pathogen. These terms deal with the biology of *Plasmodium *(including the aspects just mentioned above), and a brief section that is also in need of expansion deals with the resistance of the parasite against several anti-malarial drugs.

### IDOMAL: the vector-related contents

Although it sounds relatively easy to determine what should be included in a disease ontology, the fact that malaria is a three-organism infectious disease complicates matters to some extent. Of course, it is expected that a malaria ontology will include clinical and epidemiological concepts, and naturally all aspects of the biology of the disease are also assumed to form part of IDOMAL. But should vector biology be included or should it form an independent ontology? And if the first part of the question is answered in a positive way, to what extent should vector-related terms be included? A decision was reached to include in IDOMAL all aspects of vector biology that are crucial to malaria transmission and epidemiology. Thus, two such major components were included, insecticide resistance (IR), which is already covered by a specific ontology, MIRO [8], as well as terms pertaining to mosquito physiology. In the case of IR, clearly there is no way that all of its aspects should form an integral part of IDOMAL and it was decided to first importing from MIRO only the mechanisms of resistance as well as the actual insecticides. Therefore, terms relating to pertinent methodology and to populations were omitted from IDOMAL. It is planned, though, to later import MIRO entirely into IDOMAL, when the former ontology has been re-organized according to the BFO format. It was also chosen to omit, in the first version, terms relating to mosquito immunity [31,32] although, again, these will be included those in a future release. For the time being, for both imported classes of terms the original ID numbers that have been assigned through their inclusion in MIRO were kept, this way allowing for an unambiguous identification of the various items and avoiding later confusion. In other words, the use of both IDOMAL and MIRO by an IT tool or a database to be developed in the future would not be faced with problems of disambiguation of the terms. Similar to what was done for terms imported from MIRO, in all cases in which a term was imported from an existing ontology (e.g. physiological process are covered, in part, by the GO [33]) the original IDs were kept (see below).

A series of terms dealing with vector physiology were incorporated in IDOMAL, which in their majority concerned processes in mosquitoes that are related to transmission, directly or indirectly. Thus, larval life is only poorly addressed; in contrast, behavioural parameters such as host seeking or blood meal-related processed are described in more detail. More than 600 fully defined terms make up this part of the ontology. Table 2 lists the categories of such terms that can be found in the ontology, i.e. the upper levels of the corresponding section of IDOMAL as well as the number of terms in each one of the classes. As stated earlier, it should be noted that the number of terms indicated does not reflect unique terms, for the additional reason that certain terms that can be found as parts of different processes. Importantly, some of the processes described in the ontology don't refer to physiological processes of the vectors in a strict sense but, rather, they relate to the interactions between the vector and the vertebrate host of *Plasmodium *as well as the vector and *Plasmodium *itself. Furthermore, some of the processes (and "fiat process parts") described in IDOMAL can also be found in the GO listed as biological processes. In all cases in which a 1:1 tautology exists, the GO ID has been used to identify the terms in the malaria ontology. One needs to differentiate, though, between processes and functions, as does the GO in its division into three sub-ontologies. Therefore, IDOMAL lists a process called "cleavage by peptidase", while the GO includes the molecular function "peptidase activity". In this case, and some other similar ones, the GO term is cross-referenced but not directly imported.

As is true for the remaining IDOMAL, the terms addressing the mosquito physiological processes are certainly not exhausted, and more terms can (and will) be added in the future. This will certainly be the case when the ontology is expanded (or, potentially, entirely reorganized) to include other vector-borne diseases; virus-host interactions are, here, the best example.

Finally, a series of terms relating to vector control as such are also included in IDOMAL. It is expected that this kind of terms would be of importance given the stated possibility of malaria eradication efforts. It is worth mentioning here that throughout IDOMAL, as is the case for the malaria vertebrate host, terms that can be unambiguously linked to either vector or vector population are listed separately.

## Conclusions

The aim was to produce a tool that will be useful to the malaria community working towards effectively reducing the global malaria burden. Ontologies are such tools, as they provide the community with a common language that is equally well understood by computers and dedicated software. Thus, if/when widely accepted, ontologies provide the means to expand the information through interoperability and mutual understanding of database annotations. This possibility clearly enhances the usefulness of databases: rather than simple repositories, they advance to the level of complex tools. In spite of the fact that more than two thousand terms are included in IDOMAL, the fact that this first, working version of the malaria ontology is far from being complete has, indeed, to be emphasized. This is obviously the case with any ontology that expands and changes to satisfy advances such as scientific findings and novel ideas in any given field or domain. Moreover, mistakes and omissions are always part of such an effort, and the malaria community is invited, and urged, to provide constructive feedback. It may be a fact that in its present form, the ontology may be leaning slightly towards vectors than towards the other two key players of malaria, the vertebrate host and the parasite. An ontology is bound to constantly expand as new terms appear. Moreover, both for any expansion as well as for the optimal description of existing terms, an input from the community is a *conditio sine qua non*.

This ontology is freely available to everyone wishing to use it. The only condition linked to its usage is that, following the rules established by the OBO Foundry, if this ontology is to be changed in any sense by a user for any purpose, the name IDOMAL can no longer be used. We hope that in the near future we will be able to provide the users from the malaria community with a much better product that will greatly rely on their own criticism.

## List of Abbreviations used

BFO: Basic Formal Ontology; IDO: Infectious Disease Ontology; IT: Information tool; GO: gene Ontology; MIRO: Mosquito Insecticide Resistance Ontology; URL: Uniform Resource Locator; OBO: Open Biomedical Ontologies; NCBO: National Center for Biomedical Ontologies; CARO: Common Anatomy Reference Ontology; TGMA: Mosquito Gross Anatomy Ontology; TADS: Tick Gross Anatomy Ontology; ID: Identification Number; TRAP: thrombospondin-related anonymous protein; IR: Insecticide Resistance.

## Competing interests

The authors declare that they have no competing interests.

## Authors' contributions

PT researched and constructed a major part of the ontology, reviewed the physiological processes of the vectors, reviewed the entire ontology and discussed open questions with representatives of the community. EM researched and constructed the part of the ontology dealing with the physiological processes of the vectors. IB researched and constructed the part of the ontology dealing with malaria immunology. ElD researched and constructed the part of the ontology dealing with the vector-parasite and host-parasite interactions. EmD, ISK and MTB reviewed the ontology. CL conceived the project, obtained funding, constructed the medical part of the ontology, supervised the study and wrote the paper. All authors read, edited and approved the final manuscript.
